# Fexofenadine As Successful Adjunctive Treatment of Rheumatoid Arthritis and Trochanteric Bursitis: A Case Report

**DOI:** 10.7759/cureus.69401

**Published:** 2024-09-14

**Authors:** Ellen Yos, Emily Hines, Mary Therese Thomas, Christine M Angeles, David A Chetrit

**Affiliations:** 1 Internal Medicine, Grand Strand Medical Center, Myrtle Beach, USA; 2 Rheumatology, Carolina Health Specialists, Myrtle Beach, USA

**Keywords:** anti-histamine, disease modifying anti-rheumatic drugs (dmard), fexofenadine, rheumatoid arthritis, trochanteric bursitis

## Abstract

Rheumatoid arthritis (RA) is a chronic inflammatory joint disease that results in cartilage and bone damage, primarily involving synovial joints. The hallmark feature of this condition is inflammatory polyarthritis which can be associated with other joint pathologies, including bursitis. Many treatment options help relieve joint pain and slow down damage to the joints in both RA and bursitis. However, not all treatments are effective or affordable. These treatments include non-steroidal anti-inflammatory drugs (NSAIDs), biologic disease-modifying antirheumatic drugs (bDMARDS), conventional DMARDS (cDMARDS), and corticosteroids. This is a case of trochanteric bursitis in the setting of RA, which was subjectively and objectively treated using the histamine receptor antagonist fexofenadine.

## Introduction

Rheumatoid arthritis (RA) is an autoimmune disease mainly affecting the synovial joints, although it has the propensity to damage extra-articular organs such as the heart, lungs, and nervous system if left untreated [1]. It has become increasingly more prevalent in the population for the past three decades, with approximately 1.3 million people in the United States affected and around 1% of the population worldwide [1-3]. Although the pathogenesis of RA is multifactorial and not fully understood, it has been shown that activation of the inflammatory pathways results in tissue damage leading to pain, stiffness, tenderness, and swelling within the joints [2,3]. The disease is most prevalent in the sixth decade of life and women are affected nearly twice as often as men. A family history of RA and exposure to tobacco smoke are some of the strongest associations shown to increase the risk of developing the disease [4].

Bursitis, or inflammation of the lining of a joint space, is a common comorbidity in patients with RA [5]. The constant state of systemic inflammation in RA makes these patients prone to more frequent flares of bursitis, particularly in the hip joints [5,6]. Symptoms are successfully treated with corticosteroid injections into the affected bursa with good data for long-term remission [6]. Unfortunately for those with RA, bursal inflammation is often recurrent and can lead to long-term local steroid usage [7].

While nonpharmacologic interventions such as occupational therapy can be useful for treating the symptoms and comorbidities of RA, the most effective treatments include non-steroidal anti-inflammatory drugs (NSAIDs) and disease-modifying antirheumatic drugs (DMARDs). Both have been proven to decrease flares and can induce remission [8]. DMARDs can be delineated into two major categories: conventional and biological. Conventional DMARDs include methotrexate, leflunomide, hydroxychloroquine, and sulfasalazine. Biologic DMARDs include tumor necrosis factor α (TNFα) inhibitors, Janus kinase inhibitors, B-cell inhibitors, and interleukin inhibitors, amongst others [9].

TNFα inhibitors (TNFi) are bDMARDs that include etanercept, infliximab, and adalimumab, which block TNFα from exciting the proinflammatory pathway. Unfortunately, TNFi may also compromise the immune system and increase the risk of serious infections [10]. Therefore, these medications are typically utilized only after conventional therapy fails. Failure may be due to intolerable adverse effects or loss of drug efficacy. 

Here we present the case of a patient with poorly controlled symptoms of RA and frequent chronic trochanteric bursitis flares resulting in impairment of her quality of life. She had failed a first-line cDMARD and several bDMARDs but experienced significant relief in RA symptoms with the incidental use of fexofenadine to treat seasonal allergies. This article was previously presented as a poster at the South Atlantic Division GME Research Day 2024 on May 2, 2024.

## Case presentation

This patient was a 59-year-old female with a history of rheumatoid factor negative, cyclic citrullinated peptide positive RA diagnosed in 2013. Her RA was being treated with abatacept 125 mg subcutaneously once per week and ibuprofen 800 mg three times a day as needed. Other relevant past medical history included multisite osteoarthritis, osteoporosis, and recurrent trochanteric bursitis. The patient had previously undergone treatment with adalimumab and etanercept for her RA, both of which were discontinued due to demyelination, which manifested in the patient as numbness and tingling in the lower extremities, dizziness, and ataxia. Methotrexate was also discontinued due to loss of efficacy. While her symptoms had previously been well controlled on abatacept, she endorsed bilateral hip pain, worse on the left side, which was different from her previous sciatica but similar to her previous episodes of trochanteric bursitis.

During physical examination, there was no joint swelling, tenderness, erythema, or warmth of the trochanteric joint. There was a prominent fat pad over both ankle joints with no appreciable synovitis, but otherwise physical exam was normal. With her previous episodes of bursitis, she had variable responses to corticosteroid injections. She reported that this time she was on vacation and had taken several days’ worth of fexofenadine for allergy relief. On the days that she had taken the medication, she had noticed substantial relief of her hip pain symptoms. Of note, she was not taking any other allergy relief medications during this time, including steroids. 

**Figure 1:**
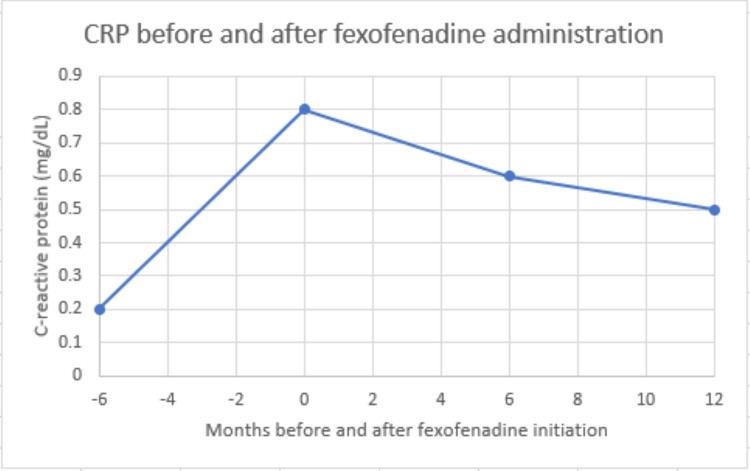
C-reactive protein before and after fexofenadine initiation. The initiation of fexofenadine occurs at the “0” month mark.

**Figure 2:**
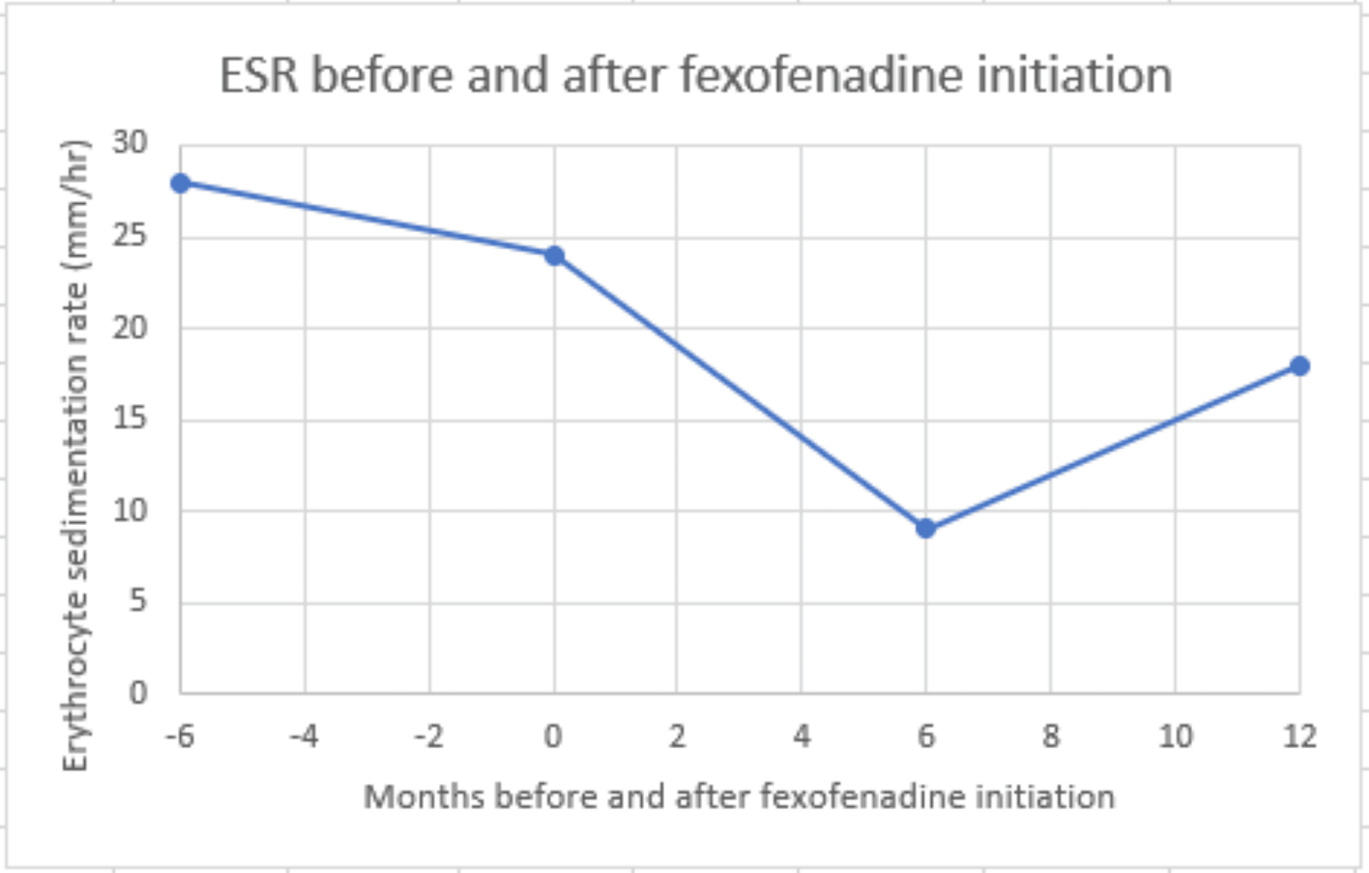
Erythrocyte sedimentation rate (ESR) before and after fexofenadine initiation. The initiation point occurs at the “0” month mark.

At a follow-up visit six months later, the patient reported that on three separate occasions, she had taken the fexofenadine when she felt a flare of her RA and bursitis. After taking the medication for two days each time, she had consistent and reproducible relief of pain. A year after the initial visit, she continued to report subjective improvement in her flares with use of the fexofenadine as needed, with one flare occurring approximately every six months. She had not required an intraarticular corticosteroid injection in over two years. She had resolved tenderness over the bilateral trochanteric bursae and subjectively improved her ability to perform her activities of daily living. Incidentally, it was noticed that once she began taking the fexofenadine, her inflammatory markers had greatly improved and remained lower than her previous flare (Figures 1, 2). The patient remains on abatacept 125 mg subcutaneously once weekly, ibuprofen 800 mg every eight hours as needed, and continues to use fexofenadine once or twice a day as needed for joint pain or seasonal allergy symptoms.

## Discussion

In the treatment of RA, methotrexate is traditionally the first-line agent. It inhibits dihydrofolate reductase, which subsequently increases the sensitivity of T cells to apoptosis. Common side effects include GI distress, hepatotoxicity, and bone marrow suppression [9]. In this patient, methotrexate was discontinued due to loss of efficacy.

In addition to methotrexate, this patient underwent frequent steroid injections for flares of trochanteric bursitis. Chronic steroid usage is linked to cortical bone thinning, spiking of blood glucose, bleeding into the joint, and occasionally infection. One particular study investigated post-menopausal women who were receiving epidural steroid injections for low back pain. After approximately 14 injections of triamcinolone at a dose of 400 mg per injection, there was a significant decrease in bone mineral density at the femoral neck and total femur [7]. This study is generalizable to our patient and demonstrates a small fraction of the risk involved in long-term steroid use.

After discontinuing methotrexate, our patient was prescribed two TNFi in succession, adalimumab and etanercept, each of which was subsequently discontinued. Adalimumab was discontinued due to lack of efficacy. Etanercept was discontinued due to TNFα-induced demyelination: a rare and unique adverse event associated with some bDMARDs.

Etanercept has been implicated in cases of post-treatment demyelination syndromes [11]. TNFi agents can worsen underlying demyelinating disease through disturbance of the TNF/TNF receptor system. As an example, the TNF system plays a role in both the pathophysiology of RA and multiple sclerosis through the overproduction of proinflammatory cytokines. It is theorized that there may be an inverse relationship between the two diseases, so that when treatment for one is initiated, it may worsen the progression of the other. After demyelination manifests, immediate discontinuation of the DMARDs results in spontaneous complete or partial resolution of the clinical symptoms [12]. In this patient, cessation of etanercept was associated with a significant improvement in her neurological symptoms. This unfortunate side effect limited the use of TNFi in her case.

The search for other effective, more cost-efficient treatments is of the utmost importance for those with RA. Of the potential candidates, fexofenadine, the third-generation antihistamine, has been brought to the forefront as one of the medications with potential effects on the TNF/TNF receptor pathway [13]. Fexofenadine primarily binds cytosolic phospholipase A2 (cPLA2), preventing the production of arachidonic acid [14]. This reduces gene expression for inflammatory cytokines encoded by TNFα, including IL-1β and IL-6.

This targeting of cPLA2 is what differentiates fexofenadine’s mechanism of action from other TNFα blockers. The targeting of cPLA2 occurs downstream in the signaling cascade, in contrast to the upstream targeting of the TNF receptor itself, which occurs with other TNFi. The cPLA2 molecule plays a large role in various autoimmune, neurodegenerative, and cardiovascular conditions, and even cancer [13]. This interaction between fexofenadine and cPLA2 provides a gateway to future discoveries and potential treatments outside of RA.

Amongst the other histamine 1 receptor 1 (H1R1) antagonists, fexofenadine has thus far proven to be unique in its effect on the TNF pathway. Liu et al. performed an in vitro and in vivo transgenic mouse study in which seven other H1R1 antagonists did not show TNFα suppression [13]. Fexofenadine was also shown to be more effective than methotrexate in preventing the inflammatory signs and symptoms of RA. In this particular case, the patient experienced fairly immediate and reproducible relief of her joint pain with infrequent use of fexofenadine.

In addition, fexofenadine has limited side effects (which may include headache, cough, drowsiness, upset stomach, and dry mouth) [15] and is sold over the counter for a significantly reduced price in comparison to DMARDs. Unfortunately, the success of the biological agents has led to a staggering financial burden on those reliant on the medications with an upward cost of $50,000 per year [16]. Contributing to the financial burden are the screening tests for tuberculosis, hepatitis B, and hepatitis C that are required prior to starting treatment with any cDMARD or bDMARD [9]. Almost all DMARDs require frequent lab work to monitor blood cell counts, renal function, and lipids, thereby increasing the cost to the patient [17].

This has led to the proposal of fexofenadine’s use for those who cannot tolerate TNFα inhibitors as an effective alternative agent [13]. The prognosis of RA is typically good; however, these patients live three to 12 years less than the general population, often resulting from increased mortality due to complications such as accelerated cardiovascular disease and rheumatoid lung disease [18]. It is imperative that research into alternative and cost-effective treatment for RA with medications like fexofenadine, such as loratadine [19] and montelukast [20], continue to be pursued.

## Conclusions

Fexofenadine may be a viable off-label option for the treatment of RA or trochanteric bursitis when traditional DMARDs and biologics are not an option. This antihistamine is proposed to have similar mechanisms of action and outcomes to DMARDs already in use, with the addition of fewer adverse events and lower financial burden.

For patients with uncontrolled RA and comorbidities such as trochanteric bursitis, clinicians should consider fexofenadine when discussing treatment options, either as adjunctive therapy or alternatives to DMARDs if medication failure occurs.
